# The Relative Efficacy of Monotherapies for Palmoplantar Pustulosis and Palmoplantar Psoriasis: A Network Meta-Analysis Study of the Palmoplantar Spectrum

**DOI:** 10.3390/medicina62020400

**Published:** 2026-02-19

**Authors:** Aditya K. Gupta, Mary A. Bamimore, Tong Wang, Tina Bhutani, Vincent Piguet, Mesbah Talukder

**Affiliations:** 1 Division of Dermatology, Temerty Faculty of Medicine, University of Toronto, Toronto, ON M5S 1A8, Canada; 2 Mediprobe Research Inc., London, ON N5X 2P1, Canada; 3 Synergy Dermatology, San Francisco, CA 94132, USA; 4 Division of Dermatology, Women’s College Hospital, Toronto, ON M5S 1B2, Canada; 5 School of Pharmacy, BRAC University, Dhaka 1212, Bangladesh

**Keywords:** palmoplantar pustulosis, palmoplantar psoriasis, biologic

## Abstract

*Background and Objectives*: Palmoplantar pustulosis (PPPust) and palmoplantar psoriasis (PPso) are distinct palm/sole dermatoses that have historically shared the abbreviation “PPP”. Though the two—since the advent of advanced biotechnology—are now deemed separate diagnoses, each still falls under the ‘palmoplantar spectrum’. It is important to note that PPso and PPPust are each distinct from generalized pustular psoriasis (GPP), a condition that is outside the scope of our study. We quantified the relative efficacy of biologic and small-molecule monotherapies on the palmoplantar spectrum using Bayesian network meta-analyses (NMAs). *Materials and Methods:* On 6 November 2025, we searched PubMed, Scopus, ClinicalTrials.gov, and citations (i.e., citation mining) for randomized trials of monotherapy reporting PPP Area and Severity Index (PPPASI) outcomes at 12 or 16 weeks; we secondarily investigated fresh pustule-related outcomes at 4 weeks. We ran Bayesian NMAs with uniform priors; nodes were defined by dose and timepoint. Interventions’ Surface Under the Cumulative Ranking Curve (SUCRA) values were computed; pairwise effects with 95% credible intervals were also estimated. Sensitivity analyses adjusted for diagnosis (pustulosis vs. psoriasis) via network meta-regression. *Results*: Twenty trials (n = 2030) with 23 active comparators provided data for 10 endpoints (fresh pustules at 4 weeks; PPPASI-50/75 and mean percentage and absolute PPPASI change at 12 and 16 weeks). *Conclusions:* The NMA indicates efficacy of ixekizumab and brodalumab (IL-17 inhibitors), guselkumab (IL-23 inhibitor), and spesolimab (IL-36 inhibitor) in managing palmoplantar pustulosis.

## 1. Introduction

Palmoplantar pustulosis and palmoplantar psoriasis are chronic dermatoses of the hand and foot that negatively impact social, occupational, and economic well-being [1]. Given that the palms and soles are principally diseased in these conditions, a diagnosis of either can lead to an inability to work (especially in roles that largely involve the use of hands, walking or standing, for example), and increased requests for sick leave, among other things. Furthermore, the overt cosmetic impact of these conditions often results in social isolation, which, in turn, can diminish the quality of life [1,2].

Though palmoplantar psoriasis and palmoplantar pustulosis have numerous similarities, the two—over the course of history—shifted from being considered synonymous to being regarded as separate entities [3,4]. Psoriasis, which was formally described as far back as the 1800s, is characterized by papulosquamous lesions that may or may not be pustular; cases of the former were a reason palmoplantar pustulosis was considered a form of psoriasis. Furthermore, this school of thought was also reflected almost a century ago in Barber’s (1933) description of a patient (whose psoriatic lesions contained pustules) [5]. Many, like Barber, assumed pustulosis was part of the psoriasis spectrum for a long time. Moreover, the interchangeability of the ‘PPP’ abbreviation with both palmoplantar pustulosis and palmoplantar psoriasis also perpetuated the amalgamation (and confusion) [6].

Since the advent of advanced biotechnology, the realization of genetic differences—which are not phenotypically obvious—created an ‘updated’ understanding of why the two dermatoses should be different [3,4,6,7]. The newer genetic evidence resulted in various agencies—including the European Rare and Severe Psoriasis Expert Network (ERASPEN) and International Psoriasis Council (IPC)—formally classifying palmoplantar pustulosis and palmoplantar psoriasis as distinct entities. While a global consensus is far from consolidated [8], many experts currently resonate with the stance of ERASPEN and IPC—and the current paper, like these two agencies, also deems the two as distinct conditions. Palmoplantar pustulosis and palmoplantar psoriasis are each different from generalized pustular psoriasis (GPP); the current paper does not address GPP.

Numerous therapeutic agents have been reported to reduce disease severity for these two dermatoses [5,6,9], and the relative efficacy of biologics and small-molecule inhibitors have been of interest to many, including Huang et al. (2024) [10], Tsiogkas et al. (2023), [11], Alshareef et al. (2025) [12], and Spencer et al. (2023) [13]. The primary objective of the current study was to determine the relative efficacy of monotherapy with biologics and small-molecule inhibitors on palmoplantar pustulosis and palmoplantar psoriasis through network meta-analyses (NMAs). Our work is primarily an analysis of the palmoplantar spectrum.

## 2. Methods

The conduct of our work was in accordance with recommendations of the Preferred Reporting Items for Systematic reviews and Meta-Analyses (PRISMA) (Supplementary Material) [14]. The current study’s protocol was registered under the Open Science Framework (OSF) platform (link: https://doi.org/10.17605/OSF.IO/AECTU), where a sample of our search strategy (for PubMed) is provided; our search was not more nor less specific than what it is to make the search and screening processes as time efficient as possible.

As mentioned earlier, the principal aim of our work was to determine the relative efficacy of monotherapy with small-molecule inhibitors and biologics on palmoplantar pustulosis and palmoplantar psoriasis; our study also had a secondary aim, which was to also determine the relative efficacy with cyclosporine, anakinra, and RIST4721 at 4 weeks.

On 6 November 2025, the peer-reviewed literature was systematically searched through PubMed and Scopus; eligible studies were—as per the PICO (i.e., Patient-Intervention-Comparator-Outcome) framework—randomized trials that investigated the efficacy of monotherapy with a biologic or small-molecule inhibitor on palmoplantar pustulosis or palmoplantar psoriasis at 12 and 16 weeks using outcome measures related to ‘PPPASI’, an abbreviation that stands for both Palmoplantar Pustulosis Area and Severity Index and Palmoplantar Psoriasis Area and Severity Index. Eligible studies were also identified through ClinicalTrials.gov and citation mining. Each eligible study’s evidence quality was assessed by using Cochrane Collaboration’s risk of bias (RoB) tools. The title, abstract, and full-text screenings of the search process were carried out independently by two authors (MAB and MT); discrepancies were resolved through discussion with a third author (AKG). The geometry of each network was depicted through a network plot (i.e., a graph of nodes and edges); a network’s geometry determined whether node-splitting analyses for inconsistency could be conducted. Furthermore, results from early phases of clinical trials would require that findings from our NMAs be interpreted cautiously.

Extracted data were organized into spreadsheets; thereafter, data were used to run Bayesian network meta-analysis with uniform priors to generate comparators’ surface under the cumulative ranking curve (SUCRA) values and pairwise relative effects. For mean changes, the pairwise relative effects were quantified as mean differences. For proportions, the pairwise relative effects were quantified as a mean difference of the log odds. Our NMAs also computed a 95% credible interval (95% CI) for each mean difference. Given our cognizance of the stance that palmoplantar pustulosis and palmoplantar psoriasis are distinct entities, we decided that our sensitivity analyses will pertain to accounting for diagnosis variation where diagnosis would be treated as an independent variable—by way of being a potential effect modifier (subgroup analysis) or confounder (covariate-adjustment). Main and sensitivity analyses were conducted using the multinma package [15] in R (version 4.3.2) via RStudio [16]. The threshold for statistical significance was 5%.

## 3. Results

**Figure 1:**
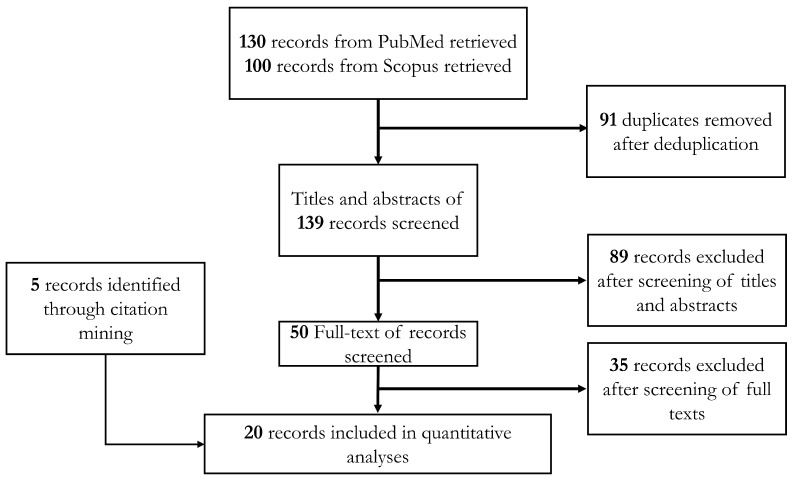
Schematic for identification of eligible studies.

The schematic for the identification of eligible studies is presented in Figure 1. Our systematic search identified 20 publications (Table 1) [17,18,19,20,21,22,23,24,25,26,27,28,29,30,31,32,33,34,35,36] and the study-level characteristics for each are provided in Table 1—where details on participants’ sex distribution, age, diagnosis, and therapeutic regimen are detailed therein. Table 2 presents details of model fit for primary outcomes as per the deviance information criterion (DIC) and effective number of parameters (pD).

**Figure 2:**
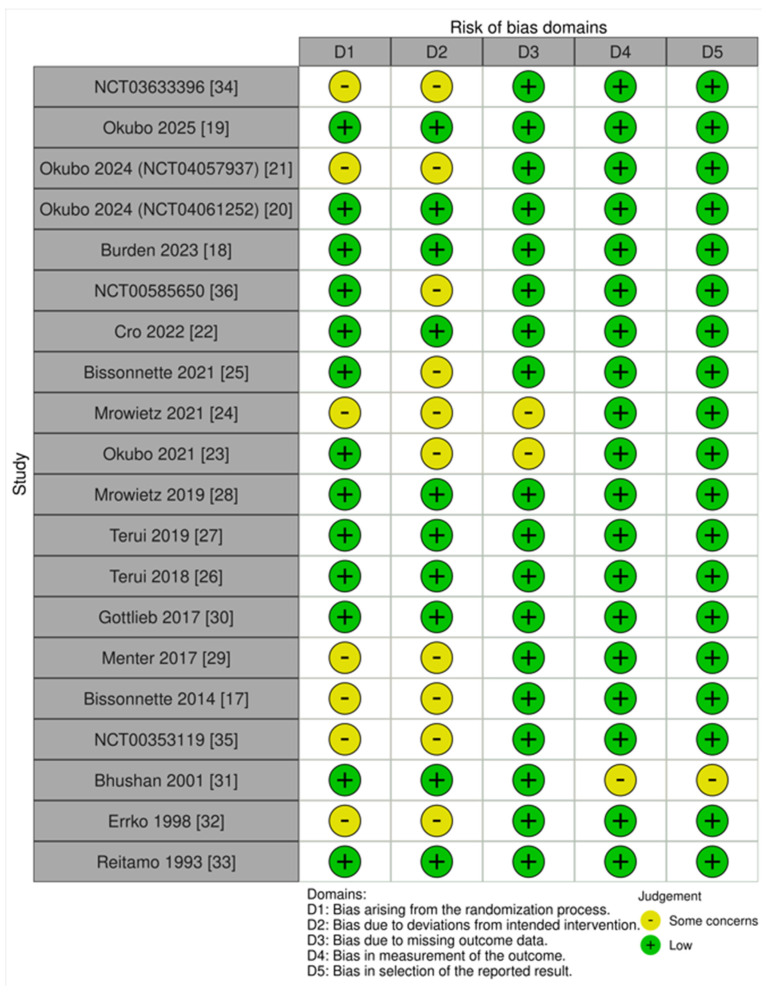
Study-level risk of bias (RoB) evaluation.

The qualitative evaluation for each eligible study’s RoB is presented in Figure 2.

Across the 20 studies that met our eligibility criteria, there were a total of 2030 participants, and approximately 45.8%, 22.9%, 20.8%, and 10.5% had a diagnosis of palmoplantar pustulosis, palmoplantar psoriasis, palmoplantar pustulosis with or without psoriasis, and palmoplantar pustular psoriasis, respectively (Table 1). We identified sufficient data to conduct analyses where nodes were defined at the level of dosage and for a singular timepoint; we had a total of 10 endpoints (i.e., outcome measures), namely, (1) mean change in number of fresh pustules at 4 weeks, (2) proportion of participants who attained at least 50% reduction in fresh pustules at 4 weeks, (3) proportion of participants who attained PPPASI 50 at 12 weeks (PPPASI-50 12 weeks), (4) proportion of participants who attained PPPASI 75 at 12 weeks (PPPASI-75 12 weeks), (5) mean percentage change (i.e., relative change) in PPPASI at 12 weeks, (6) mean absolute change in PPPASI at 12 weeks, (7) proportion of participants who attained PPPASI 50 at 16 weeks (PPPASI-50 16 weeks), (8) proportion of participants who attained PPPASI 75 at 16 weeks (PPPASI-75 16 weeks), (9) mean percentage change (i.e., relative change) in PPPASI at 16 weeks, and (10) mean absolute change in PPPASI at 16 weeks. The network plots for all outcomes are presented in Appendix A of the Supplement. The geometry of the network precluded the conduct of node-splitting analysis of inconsistency (i.e., there were no closed loops in any of the networks) [37].

**Figure 3:**
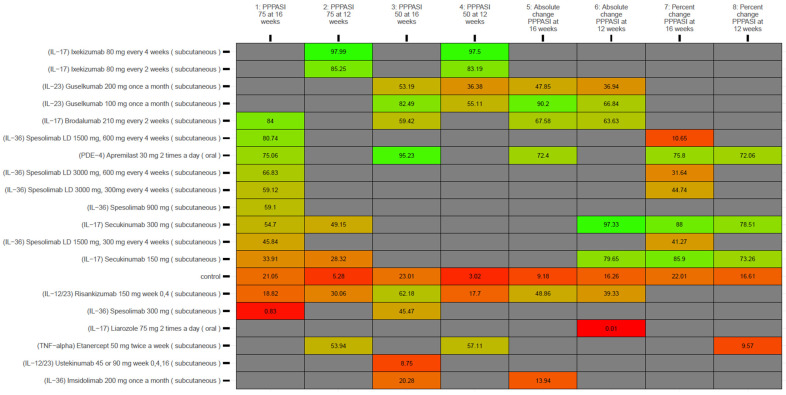
Kilim plot for comparators’ surface under the cumulative ranking curve (SUCRA) values under base model (i.e., main analyses), for the 12- and 16-week. Outcomes related to PPPASI. The SUCRA value for each comparator represents its overall efficacy ranking, with higher values indicating a more favorable outcome. The vertical axis lists all comparators, consisting of 19 active treatments and one inactive control (the placebo/vehicle node). The horizontal axis shows the evaluated endpoints from main analyses, with SUCRA values expressed as percentages. Red and green boxes (chosen arbitrarily) indicate the lowest and highest SUCRA values, respectively. Greener shading denotes higher effectiveness, while redder shading denotes lower effectiveness. Grey boxes indicate that no data were available. This visualization highlights how treatments compare, from least to most effective, according to this metric.

**Figure 4:**
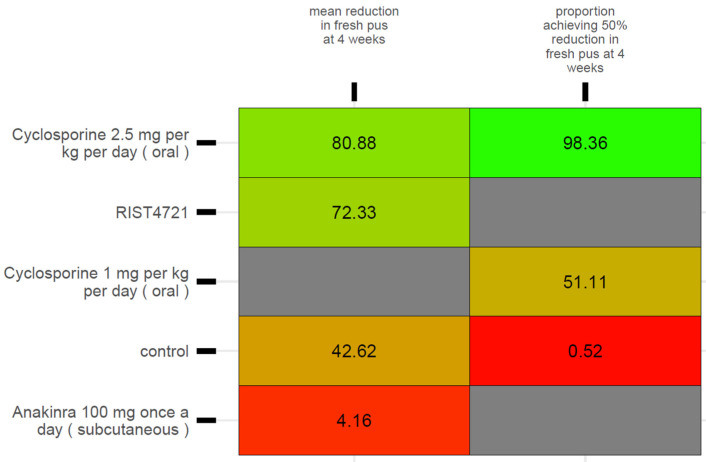
Kilim plot for comparators’ surface under the cumulative ranking curve (SUCRA) values, under the base model, for the 4-week outcomes. The SUCRA value for each comparator represents its overall efficacy ranking, with higher values indicating a more favorable outcome. The vertical axis lists all comparators, consisting of 19 active treatments and one inactive control (the placebo/vehicle node). The horizontal axis shows the evaluated endpoints from main analyses, with SUCRA values expressed as percentages. Red and green boxes (chosen arbitrarily) indicate the lowest and highest SUCRA values, respectively. Greener shading denotes higher effectiveness, while redder shading denotes lower effectiveness. Grey boxes indicate that no data were available. This visualization highlights how treatments compare, from least to most effective, according to this metric.

Comparators’ SUCRA values for the 12-week and 16-week outcomes—as per the base NMA (i.e., main model) and for the palmoplantar spectrum—are presented in Figure 3. Comparators’ SUCRA values under the diagnosis-adjusted (i.e., covariate-adjusted) sensitivity analyses are presented along with SUCRA values from the base model in Appendix B of the Supplement, and Figure 4 presents comparators’ SUCRA values at 4 weeks as per the base model.

**Figure 5:**
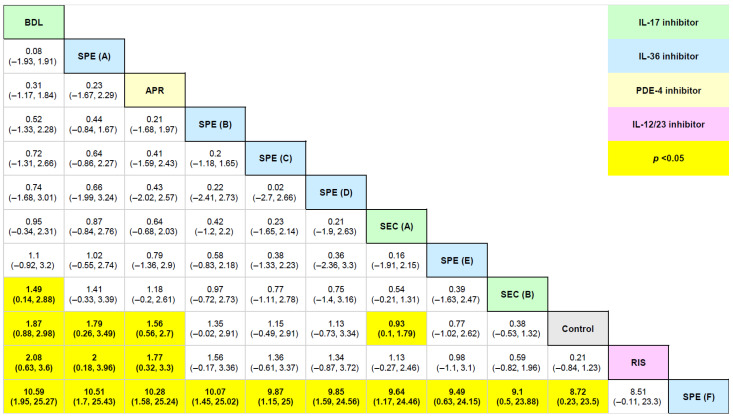
League table for PPPASI 75 16 weeks. League tables are visual tools to depict the relative efficacy for every possible pairwise combination of comparators. Abbreviations: APR, apremilast 30 mg 2 times a day (oral); BDL, brodalumab 210 mg every 2 weeks (subcutaneous); RIS, risankizumab 150 mg at weeks 0, 4 (subcutaneous); SEC (A), secukinumab 300 mg (subcutaneous); SEC (B), secukinumab 150 mg (subcutaneous); SPE (A), spesolimab LD 1500 mg, 600 mg every 4 weeks (subcutaneous); SPE (B), spesolimab LD 3000 mg, 600 mg every 4 weeks (subcutaneous); SPE (C), spesolimab LD 3000 mg, 300 mg every 4 weeks (subcutaneous); SPE (D), spesolimab 900 mg (subcutaneous); SPE (E), spesolimab LD 1500 mg, 300 mg every 4 weeks (subcutaneous); SPE (F), spesolimab 300 mg (subcutaneous).

Across the 20 eligible studies, 23 active comparators were identified, namely, (1) anakinra 100 mg once a day (subcutaneous), (2) apremilast 30 mg 2 times a day (oral), (3) brodalumab 210 mg every 2 weeks (subcutaneous), (4) cyclosporine 1 mg per kg per day (oral), (5) cyclosporine 2.5 mg per kg per day (oral), (6) etanercept 50 mg twice a week (subcutaneous), (7) guselkumab 100 mg once a month (subcutaneous), (8) guselkumab 200 mg once a month (subcutaneous), (9) imsidolimab 200 mg once a month (subcutaneous), (10) ixekizumab 80 mg every 2 weeks (subcutaneous), (11) ixekizumab 80 mg every 4 weeks (subcutaneous), (12) liarozole 75 mg 2 times a day (oral), (13) risankizumab 150 mg week 0,4 (subcutaneous), (14) RIST4721 300 mg once a day (oral), (15) secukinumab 150 mg (subcutaneous), (16) secukinumab 300 mg (subcutaneous), (17) spesolimab 300 mg (subcutaneous), (18) spesolimab 900 mg (subcutaneous), (19) spesolimab LD 1500 mg, 300 mg every 4 weeks (subcutaneous), (20) spesolimab LD 1500 mg, 600 mg every 4 weeks (subcutaneous), (21) spesolimab LD 3000 mg, 300mg every 4 weeks (subcutaneous), (22) spesolimab LD 3000 mg, 600 mg every 4 weeks (subcutaneous), and (23) ustekinumab 45 or 90 mg week 0, 4, 16 (subcutaneous) (Table 1). The pairwise relative effects for PPPASI-75 16 weeks, the network with the largest number of comparators, are presented in the league table Figure 5. The pairwise relative effects for all the other outcomes are presented in Appendix C of the Supplement.

## 4. Discussion

We conducted an NMA study to determine the relative efficacy of monotherapy with biologics and small-molecule inhibitors for the palmoplantar spectrum in terms of 10 clinically relevant outcomes; we conducted additional analyses that accounted for the specific diagnosis, as PPso and PPPust are still distinct conditions which fall under the palmoplantar spectrum.

**Figure 6:**
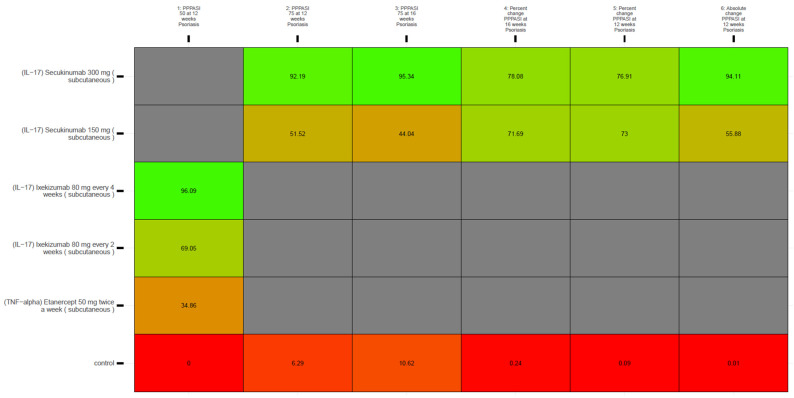
Kilim plot for comparators’ surface under the cumulative ranking curve (SUCRA) values, for the subgroup of participants diagnosed with palmoplantar psoriasis only. The SUCRA value for each comparator corresponds to its ranking for efficacy—where higher values represent a more favorable outcome. On the vertical axis are all comparators; we identified 5 active treatments and one inactive control (the placebo/vehicle node). The horizontal axis represents the endpoints, and SUCRA values—expressed as percentages—are presented in cells. Red and green boxes (chosen arbitrarily) indicate the lowest and highest SUCRA values, respectively. Greener shading denotes higher effectiveness, while redder shading denotes lower effectiveness. Grey boxes indicate that no data were available.

We identified 20 publications that met our eligibility criteria—and across which were a total of 2030 participants. Given that the current literature supports the two palmoplantar dermatoses as separate diagnoses, part of our quantitative syntheses involved accounting for the distinct entities. Diagnosis-wise, the largest subgroup of dermatoses was palmoplantar pustulosis (approximately 45.8%). The psoriasis-only (i.e., palmoplantar psoriasis) subgroup constituted 22.9% of the 2030 participants; we also determined the relative efficacy of monotherapies in this subgroup, and the resulting SUCRA values are presented in the kilim plot in Figure 6.

We acknowledge that a main limitation of our work is the inability to adequately assess transitivity given networks’ geometry and small sample sizes. Independent of network geometry and sample size, study-level variation in potential effect modifiers—such as disease severity at baseline and smoking status—could not be accounted for, as such information was not provided by all studies using consistent metrics. For example, participants’ smoking status at baseline was not provided in the paper by Menter et al. (2017) [29]—while it was in the papers by Mrowietz et al. (2021) [24] and Okubo et al. (2025) [19]. Hence, any interpretation of the current study’s results should be cautiously made in light of the limitations. Furthermore, analyses based on results from an early phase of clinical trials are another reason to interpret results cautiously.

A strength of our comparative analysis is the level of stringency with which outcome measures were defined. Each outcome measure was for a specific (i.e., singular) timepoint—unlike previous NMA studies, such as the one by Huang et al. (2024) [10], where 12-week and 16-week outcome data were pooled into a single analysis for percentage change in PPPASI.11 Furthermore, each node corresponded to a specific dose (or dosage) in our study; this is why outcome data from ixekizumab 80 mg every 2 weeks (subcutaneous) and ixekizumab 80 mg every 4 weeks (subcutaneous) were not amalgamated in our analyses.

Another strength of the current work is the choice of sensitivity analyses. For sensitivity analyses, we accounted for variation in the disease condition in two ways, namely, by way of covariate adjustment and subgroup analyses. In covariate adjustment, we ran a network meta-regression where the analysis treated the diagnosis as a categorical variable; in subgroup analysis, only the subgroup of palmoplantar pustulosis was analyzed. Sensitivity analyses were conducted only for the 12-week and 16-week outcomes; we did not perform covariate adjustment, nor subgroup analyses, for the 4-week outcomes because all participants across those networks shared one diagnosis (i.e., palmoplantar pustulosis). For each of the 12-week and 16-week outcomes, we found that both covariate adjustment and subgroup analyses substantiated the findings from our base model. It should be noted that the conduct of these sensitivity analyses cannot be conflated with the transitivity assumption being upheld because, as mentioned earlier, diagnoses subgroup is independent of other potential effect modifiers like smoking status and disease severity at baseline.

Palmoplantar pustulosis has a female predominance [38,39] and age of onset that ranges anywhere between 40 and 60 years [39]. These were reflected in the current NMA study, because the majority (i.e., 67%) of the 2030 participants across our eligible studies were female. Across the eligible studies, the trials with the lowest and highest mean ages (in years) were, respectively, 40.8 (±11.49) and 61.33 (±24.8). However, we did not adjust for variation in gender distribution because the systematic review by Hsu et al. (2025) [40] suggests that this female predominance is far from universal, as some Asian countries show a higher male preponderance. Furthermore, the female/male ratio of palmoplantar pustulosis may actually be—according to Hsu et al. (2025)—attributable to (or confounded by) smoking status, which many have stated to be a risk factor for developing the condition [38,39,40]. Many trial studies on palmoplantar pustulosis—including those that were incorporated into the current NMA study—did not report smoking status; thus, future trials should routinely collect data on smoking status to make future quantitative syntheses much more informative.

Though we had acknowledged the need for caution when making inferences from the current study’s results, conclusions from the current study—despite limitations—tally with previous works. For palmoplantar pustulosis, our results are congruent with previous studies. For the analysis of PPPASI 75, Alshareef et al. (2025) [12] did not differentiate timepoints (i.e., all timepoints were amalgamated). Notwithstanding that, we also—like Alshareef et al. (2025) [12]—found spesolimab 900 mg (subcutaneous) to be ranked higher than secukinumab 300 mg (subcutaneous). Like Alshareef et al. [12], we found secukinumab 300 mg (subcutaneous) to be ranked higher than secukinumab 150 mg (subcutaneous). For PPPASI 50, like Alshareef et al. (2025) [12], our NMA also ranked guselkumab 100 mg once a month (subcutaneous) higher than guselkumab 200 mg once a month (subcutaneous).

Though results of our analyses are far from robust, our findings still update the results of previous NMA studies by Huang et al. (2024) [10], Tsiogkas et al. (2023) [11], Alshareef et al. (2025) [12], and Spencer et al. (2023) [13] by providing comparative evidence for regimens that have never been—so far—published for this condition, namely, brodalumab 210 mg every 2 weeks (subcutaneous) (IL-17 inhibitor), and risankizumab 150 mg week 0, 4 (subcutaneous) (IL 12/23 inhibitor). Brodalumab has been approved for treating PPP in Japan [41].

Nam et al.’s (2025) [41] study reported that IL 17 and IL 36 inhibitors demonstrate mixed results. Our findings also reflect this. For instance, liarozole (IL-17 inhibitor) was ranked low for efficacy while ixekizumab (IL-17 inhibitor) was ranked high for efficacy. Similarly, one of the regimens with spesolimab (IL-36 inhibitor) was ranked high for efficacy, while imsidolimab (IL-36 inhibitor) was ranked low for efficacy. Some studies suggest that apremilast (PDE-4 inhibitor) can be effective.

In contrast, the most efficacious agents for managing palmoplantar plaque psoriasis are secukinumab and ixekizumab (IL-17 inhibitors) (Figure 6). Our findings for the psoriasis subgroup tallies with those of Huang et al. (2024) [10] who also found ixekizumab to be ranked most efficacious for a 75% (or greater) improvement in palmoplantar score at 12–16 weeks [10].

As mentioned earlier, the current body of evidence concludes that palmoplantar psoriasis and palmoplantar pustulosis are genetically—and thereby pathophysiologically—distinct; hence, therapeutic agents’ mechanism of action between these two dermatoses would be—intuitively—distinct [42,43]. While the literature is yet to fully resolve the nuances in the biologic agents’ mechanism of action between the two conditions, various studies support some biologics being better suited to treat either condition. Through multiple genetic experiments (including RNA-sequencing profiling, proteomic analysis and histologic assessments), Wang et al. (2023) [44] found that gene expression of IL-36 was positively correlated with palmoplantar pustular psoriasis—while negatively correlated with palmoplantar psoriasis (i.e., non-pustular palmoplantar psoriasis).

Furthermore, the authors also found that genetic expression of IL-23 and IL-17 was higher in palmoplantar psoriasis (i.e., non-pustular palmoplantar psoriasis) than in subjects with palmoplantar pustular psoriasis. Our quantitative syntheses corroborate Wang et al.’s (2023) experimental analyses as we found IL-17 inhibitors to be ranked most efficacious for the psoriasis-only (i.e., palmoplantar psoriasis only) subgroup (Figure 6).

Findings from our analyses collectively allude to IL-36 inhibition being a suitable therapeutic option for conditions with pustulosis involvement; for instance, spesolimab (an IL-36 inhibitor) was the second most efficacious option for our network corresponding to PPPASI 75 at 16 weeks—wherein 542 of the 779 (i.e., almost 70%) participants had pustulosis involvement). Our work highlights the salience of conducting larger efficacy trial studies for the various classes of agents, as more studies will resolve inconsistent evidence. Findings from our work also serve as a rationale for monitoring drugs’ adverse events—especially with the publication of case reports such as the one by Jayasekera et al. (2014) [45]. The authors reported a case where PPP diagnosis was an adverse event of (i.e., secondary to) the prescription of an anti-tumor necrosis factor agent. This case of PPP was resolved through therapy with tocilizumab, interleukin-6 blocker.

Results from analyses underscore the relevance of future trials demarcating between disease conditions in their inclusion/exclusion criteria to aid syntheses studies—such as ours—in producing more clinically relevant findings.

## Figures and Tables

**Table 1:** Descriptive study-level summary data of eligible studies.

Author	Diagnosis	Agent	Regimen	Route	Sample Size at Baseline	Age	±SD	Male	Female
NCT03633396 [34]	Palmoplantar pustulosis	Imsidolimab	200 mg once a month	Subcutaneous	30	52.3	12.1	8	22
NCT03633396 [34]	Palmoplantar pustulosis	Placebo			29	47.7	10.59	5	24
2. Okubo 2025 [19]	Palmoplantar pustulosis	Placebo			58	56.4	11.2	8	50
Okubo 2025 [19]	Palmoplantar pustulosis	Risankizumab	150 mg week 0, 4	Subcutaneous	61	54.4	10.6	17	44
3. Okubo 2024 [21] (NCT04057937)	Palmoplantar pustulosis	Apremilast	30 mg 2 times a day	Oral	46	54.9	11.3	10	36
Okubo 2024 [21] (NCT04057937)	Palmoplantar pustulosis	Placebo			44	54.7	11.68	11	33
4. Okubo 2024 [20] (NCT04061252)	Palmoplantar pustulosis	Brodalumab	210 mg every 2 weeks	Subcutaneous	63	54.2	9	9	54
Okubo 2024 [20] (NCT04061252)	Palmoplantar pustulosis	Placebo			62	53.9	11	10	52
5. Burden 2023 [18]	Palmoplantar pustulosis with or without psoriasis	Placebo			43	57.7	10.1	8	35
Burden 2023 [18]	Palmoplantar pustulosis with or without psoriasis	Spesolimab	LD 1500 mg, 300 mg every 4 weeks	Subcutaneous	22	54.2	12.3	7	15
Burden 2023 [18]	Palmoplantar pustulosis with or without psoriasis	Spesolimab	LD 1500 mg, 600 mg every 4 weeks	Subcutaneous	21	51.6	7.9	5	16
Burden 2023 [18]	Palmoplantar pustulosis with or without psoriasis	Spesolimab	LD 3000 mg, 300mg every 4 weeks	Subcutaneous	22	52.8	9.2	5	17
Burden 2023 [18]	Palmoplantar pustulosis with or without psoriasis	Spesolimab	LD 3000 mg, 600 mg every 4 weeks	Subcutaneous	44	53.4	13	17	27
6. NCT00585650 [36]	Palmoplantar psoriasis	Etanercept	50 mg twice a week	Subcutaneous	8			3	5
NCT00585650 [36]	Palmoplantar psoriasis	Placebo			12			3	9
7. Cro 2022 [22]	Palmoplantar pustulosis with or without psoriasis	Anakinra	100 mg once a day	Subcutaneous	31	49.9	11.9	4	27
Cro 2022 [22]	Palmoplantar pustulosis with or without psoriasis	Placebo			33	51.7	13.6	6	27
8. Bissonnette 2021 [25]	Palmoplantar pustulosis	Placebo			19	50	12.27	3	16
Bissonnette 2021 [25]		RIST4721	300 mg once a day	Oral	15	50.5	11.22	3	12
9. Mrowietz 2021 [24]	Palmoplantar pustulosis with or without psoriasis	Placebo			21	46.3	11.7	4	17
Mrowietz 2021 [24]	Palmoplantar pustulosis with or without psoriasis	Spesolimab	900 mg	Subcutaneous	19	49.4	11.3	3	16
Mrowietz 2021 [24]	Palmoplantar pustulosis with or without psoriasis	Spesolimab	300 mg	Subcutaneous	19	54.6	7.7	3	16
10. Okubo 2021 [23]	Palmoplantar pustulosis	Guselkumab	100 mg once a month	Subcutaneous	54	53.9	10.88	8	46
Okubo 2021 [23]	Palmoplantar pustulosis	Guselkumab	200 mg once a month	Subcutaneous	52	52.9	13.39	16	36
Okubo 2021 [23]	Palmoplantar pustulosis	Placebo			53	53	9.49	9	44
11. Mrowietz 2019 [28]	Palmoplantar pustular psoriasis	Placebo			78	52.9	11.3	19	59
Mrowietz 2019 [28]	Palmoplantar pustular psoriasis	Secukinumab	300 mg	Subcutaneous	79	50.6	14.8	15	64
Mrowietz 2019 [28]	Palmoplantar pustular psoriasis	Secukinumab	150 mg	Subcutaneous	80	50.7	13.7	17	63
12. Terui 2019 [27]	Palmoplantar pustulosis	Guselkumab	100 mg once a month	Subcutaneous	54	53.9	10.88	8	46
Terui 2019 [27]	Palmoplantar pustulosis	Guselkumab	200 mg once a month	Subcutaneous	52	52.9	13.39	16	36
Terui 2019 [27]	Palmoplantar pustulosis	Placebo			53	53	9.49	9	44
13. Terui 2018 [26]	Palmoplantar pustulosis	Guselkumab	200 mg once a month	Subcutaneous	25	52 †	28–67 †	7	18
Terui 2018 [26]	Palmoplantar pustulosis	Placebo			24	52 †	32–77 †	7	17
14. Gottlieb 2017 [30]	Palmoplantar psoriasis	Placebo			68	50.9	13	34	34
Gottlieb 2017 [30]	Palmoplantar psoriasis	Secukinumab	300 mg	Subcutaneous	69	48.4	14.2	38	31
Gottlieb 2017 [30]	Palmoplantar psoriasis	Secukinumab	150 mg	Subcutaneous	68	52.4	12.6	40	28
15. Menter 2017	Palmoplantar psoriasis	Etanercept	50 mg twice a week	Subcutaneous	59	50.2	12.7	43	16
Menter 2017 [29]	Palmoplantar psoriasis	Ixekizumab	80 mg every 4 weeks	Subcutaneous	92	46.4	12.5	68	24
Menter 2017 [29]	Palmoplantar psoriasis	Ixekizumab	80 mg every 2 weeks	Subcutaneous	114	48.9	12.6	74	40
Menter 2017 [29]	Palmoplantar psoriasis	Placebo			85	49.5	12.3	64	21
16. Bissonnette 2014 [17]	Palmoplantar pustular psoriasis	Placebo			10	50.5	8.13	2	8
	Palmoplantar pustular psoriasis	Ustekinumab	45 or 90 mg week 0, 4, 16	Subcutaneous	10	55.3	6.75	1	9
	Palmoplantar pustulosis	Placebo			8	52	9.37	3	5
	Palmoplantar pustulosis	Ustekinumab	45 or 90 mg week 0, 4, 16	Subcutaneous	5	49.8	8.41	0	5
17. NCT00353119 [35]	Palmoplantar psoriasis	Etanercept	50 mg twice a week	Subcutaneous	10	46.9	13.3	1	9
	Palmoplantar psoriasis	Placebo			5	54.2	4.76	0	5
18. Bhushan 2001 [31]	Palmoplantar pustulosis	Liarozole	75 mg 2 times a day	Oral	7	63†	47–74 †	2	5
	Palmoplantar pustulosis	Placebo			8	58.5†	42–63 †	1	7
19. Erkko 1998 [32]	Palmoplantar pustulosis	Cyclosporine	1 mg per kg per day	Oral	15	45.2	12.1	2	13
	Palmoplantar pustulosis	Placebo			31	43	13.3	12	19
20. Reitamo 1993 [33]	Palmoplantar pustulosis	Cyclosporine	2.5 mg per kg per day	Oral	20	40.8	11.49	6	14
	Palmoplantar pustulosis	Placebo			20	41.65	10.41	6	14

Abbreviations: ±SD = standard deviation (for the mean age) mg = milligrams. Notes: † Terui et al. (2018) reported the median and range for age; using this median and range data, we calculated the mean age (±SD) for the guselkumab and placebo arms—which were, respectively, 49 (±30.66) and 53.67 (±35.46). † Bhushan et al. (2001) reported the median and range for age; using this median and range data, we calculated the mean age (±SD) for the liarozole and placebo arms—which were, respectively, 61.33 (±24.8) and 54.5 (±18.76).

**Table 2:** Model diagnostics of network for primary outcomes.

	12 Weeks				16 Weeks			
Outcome	Percent Change in PPPASI	Absolute Change in PPPASI	PPPASI 50	PPPASI 75	Percent Change in PPPASI	Absolute Change in PPPASI	PPPASI 50	PPPASI 75
Residual deviance	13.7	15.8	17.4	9.1	17.2	11.8	23.3	16.3
pD	5.3	13.8	10.1	9.1	6.9	11.7	18.5	16.1
DIC	19	29.6	27.5	18.2	24.1	23.5	41.8	32.4

Abbreviations: DIC = deviance information criterion, pD = effective number of parameters.

## Data Availability

The raw data supporting the conclusions of this article will be made available by the authors on request.

## Supplementary Materials

The following supporting information can be downloaded at https://www.mdpi.com/article/10.3390/medicina62020400/s1, Table S1: PRISMA_2020_checklist.
